# Experience With Normal Breathhold Planning Scans for Radiosurgery of Moving Targets With Live Tracking

**DOI:** 10.7759/cureus.30676

**Published:** 2022-10-25

**Authors:** Jimm Grimm, Shiva F Naidoo, Kristena Yossef, Gaurav Shukla, Carla J Scofield, Abby C Searfoss, Madison A Zulkoski, James A Tanyi, Heath B Mackley, Anand Mahadevan

**Affiliations:** 1 Radiation Oncology, Geisinger Cancer Institute, Danville, USA; 2 Internal Medicine, Geisinger Cancer Institute, Danville, USA

**Keywords:** real-time tracking, fiducial markers, normal breathhold, 4dct, motion management, synchrony tracking

## Abstract

Purpose: Utilization of breathhold scans with live tracking has a long track record of good published outcomes for stereotactic body radiation therapy (SBRT) and is recommended by the manufacturer of the Synchrony tracking system. However, the popularity of four-dimensional computed tomography (4DCT) scans challenges the validity of the breathhold scan with live tracking technique. Although this study is not intended to prove the superiority of either method, we demonstrate the feasibility of using the breathhold scans with a phantom test and clinical examples.

Methods: A 4DCT of a perfect sphere was scanned at 20 breaths per minute and compared to a 4DCT of a small lung tumor in one patient and a 4DCT of a larger renal tumor in another patient, as well as to fiducial matching in a patient with pancreatic cancer. Normal exhale and normal inhale breathhold CT scans were performed for the pancreatic cancer patient, combined with Synchrony tracking on CyberKnife (Sunnyvale, CA: Accuray) for treatment.

Results: The 4DCT scan of the phantom exhibited considerable apparent deformation, which must be entirely due to imaging artifact since the perfect sphere in the phantom is known to be completely rigid. The 4DCT of the lung and renal tumors in patients had similar apparent deformation. Usually in patients, from 4DCT alone, it is difficult to determine how much was due to deformation and how much was due to artifact. Fiducial positions in the final normal exhale and normal inhale breathhold scans for Synchrony matched each other within 1mm for the pancreatic cancer patient.

Conclusion: We demonstrated the feasibility of breathhold scans with Synchrony live tracking, as recommended by the manufacturer. More studies will be needed to determine whether this method is better than using a 4DCT.

## Introduction

Synchrony live tracking is available on both CyberKnife and Radixact (Sunnyvale, CA: Accuray) [1]. When live tracking is used to account for free-breathing tumor motion during treatment, it is possible to use normal (e.g., not forced) breathhold computed tomography (CT) scans for treatment planning [2]. Synchrony rigidly tracks the fiducials, so only a single exhale scan is required for the treatment plan [2]. However, it is important to confirm that the tumor does not deform significantly while breathing, which can be done by comparing fiducial locations in two normal exhale breathhold scans to a normal inhale breathhold scan that have been fused together [3]. It can usually be confirmed that deformation of the tumor in the region of the fiducials is less than a millimeter since many tumors have been measured to deform less than a millimeter during treatment [4]. The breathhold technique is recommended by the manufacturer of the Synchrony system and excellent outcomes have been achieved with this technique [5]; nevertheless, some Synchrony users are trying four-dimensional CT (4DCT) because of its widespread usage throughout the field. This study is not intended to prove superiority of either one, but rather just to explain the methodology of the breathhold technique and present results in a phantom and in a few patients.

## Materials and methods

**Figure 1:**
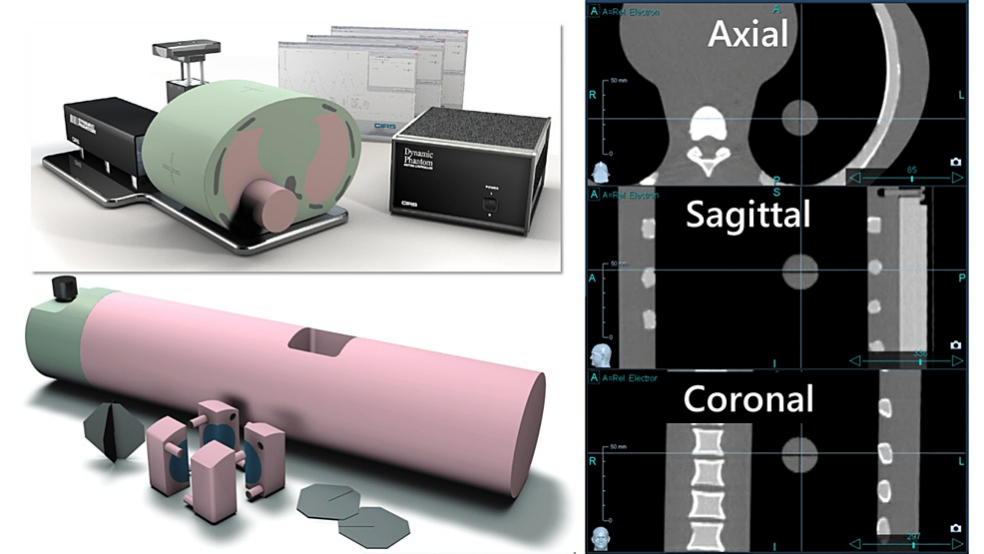
The programmable Dynamic Thorax Phantom with interchangeable inserts contains rigid lung motion phantom showing a perfectly spherical 25mm simulated lung tumor. Dynamic Thorax Phantom, model 18023-A (CIRS Inc.: Norfolk, VA)

The Dynamic Thorax Phantom, model 18023-A (Norfolk, VA: CIRS Inc.) contains a 25mm rigid spherical target inside a lung phantom motion cylinder, designed for quality assurance (QA) of the Synchrony system (Sunnyvale, CA: Accuray) (Figure 1). The normal respiration rate for an adult at rest is 12-20 breaths per minute (BPM) [6], so to ensure acceptable quality up to the high end of normal patient breathing rates, our tests were set to about 20 BPM; which is the same speed we usually use for Synchrony monthly QA. Both the 4DCT and the breathhold CT scans used 1.25mm thick slices on a 16-slice scanner. A 10-phase 4DCT was acquired for the lung phantom and for patient scans of a small lung tumor and a larger renal tumor.

For the breathhold scans of a pancreatic cancer patient, the real-time position management (RPM) system (Varian: Palo Alto, CA) was used to visualize the patient’s breathing waveform to determine the extent of normal breathing. The breathhold patient was instructed to hold their breath in a normal exhale and then normal inhale position, and both positions were practiced several times until the patient was able to hold their breath near the marks set in the RPM software; not with any submillimeter requirement, but just within sufficiently large exhale and inhale positions to assess any potential normal breathing deformation. Then, timed with contrast injection and uptake of tri-phase contrast, we performed two normal exhale scans followed by a normal inhale scan (if a scan with dense contrast is used as the planning CT it may be advisable to override the density for dose calculation. If contrast is dense enough to be visible in the digitally reconstructed radiographs (DRRs) then it might not be usable as the planning CT since it could affect the targeting accuracy, especially for non-fiducial tracking modes). Our scanner is configured to automatically reconstruct with metal artifact reduction (MAR) and automatically send to the planning system, so the physics and dosimetry team can already begin fusing each scan as it comes in, while therapists are finishing subsequent breathhold phases and taking the setup photos and discussing the upcoming treatment with the patient. While the patient waited on the table, a quick preliminary fusion of the breathhold scans was performed to ensure the fiducials agreed in all the breathhold phases. If any scans were mismatched, they were repeated until a set of three breathhold scans were in acceptable agreement.

## Results

**Figure 2:**
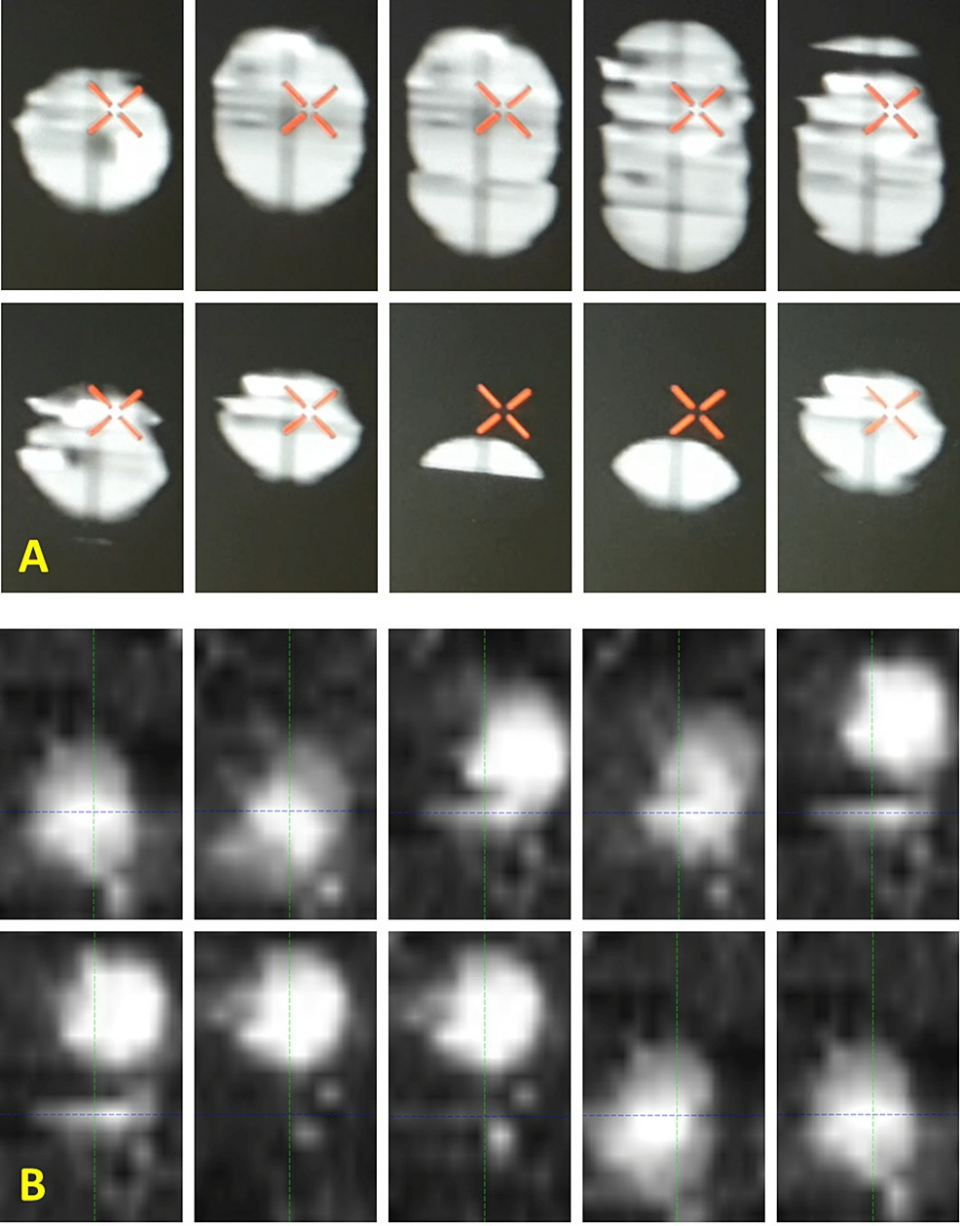
Spherical target and lung tumor near the diaphragm. (A) Sagittal view of spherical target from Figure 1 scanned as a 4DCT with 10 phases. Since the target is known to be a rigid perfect sphere, the apparent deformation is known to be entirely artifact from the 4DCT. None of these 10 phases are suitable for submillimeter end-to-end tracking. (B) Sagittal view of a 2-3cm lung tumor near the diaphragm. In a patient, it is harder to differentiate deformation from artifact, but the tumor does not separate and re-join as it appears to in the 4DCT, so much of the apparent deformation is probably 4DCT artifact. This scan was not used to treat the patient.

The sphere in the phantom of Figure 1 appeared to have a large amount of deformation in the 4DCT in Figure 2A, even separating and rejoining, but since the sphere is known to be rigid this must be due to 4DCT artifact. The lung tumor in Figure 2B appears to have deformation, but since it is in a patient, it is harder to be sure whether it is truly tumor deformation or 4DCT artifact. However, since a few slices of the lung tumor are separated from the rest in some phases and re-joined with the tumor in other phases, it is likely that at least a large portion of the visible distortion is due to 4DCT artifact.

**Figure 3:**
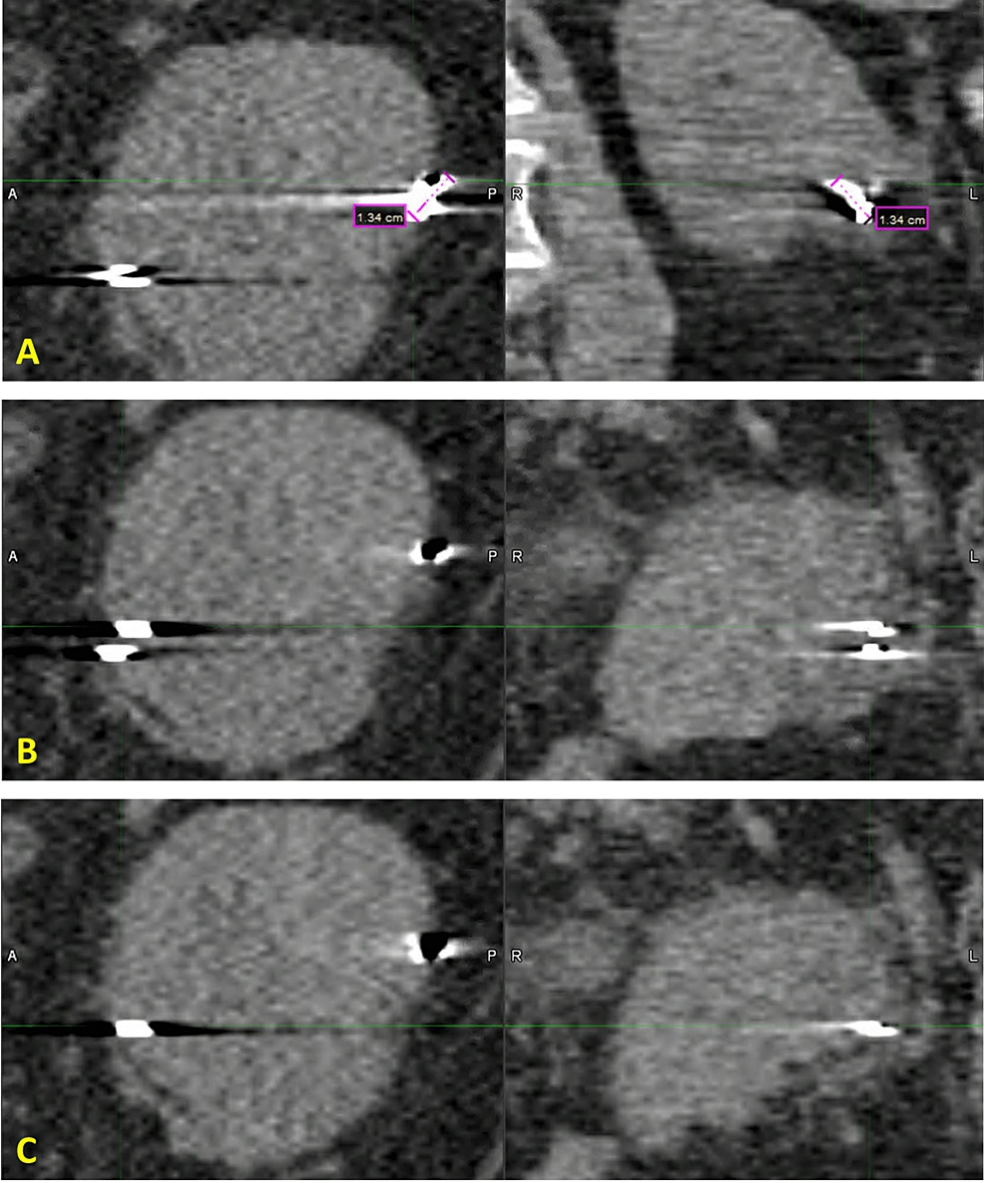
Renal case study 4DCT, sagittal and coronal views. (A) In the 80% phase, one fiducial appears to be 13mm long but this is more than double the known length of 5mm, suggesting it is from 4DCT artifact. (B) In the 90% phase, one fiducial appears to be two fiducials, but in the 80% phase (A) and 0% phase (C), it is confirmed to be a single fiducial; therefore it must be due to 4DCT artifact. This scan was not used in treatment planning. 4DCT: four-dimensional computed tomography

One of the fiducials in the 80% phase of the 4DCT of the renal tumor in Figure 3A appeared to be 13mm long, which is likely due to 4DCT artifact, since the fiducial is known to be 5mm long. In Figure 3B, a fiducial is separated into two parts in the 90% phase, but it is clearly a single fiducial as seen in the 80% and 0% phases in Figures 3A-3C, suggesting that this also must be an effect of the 4DCT artifact.

**Figure 4:**
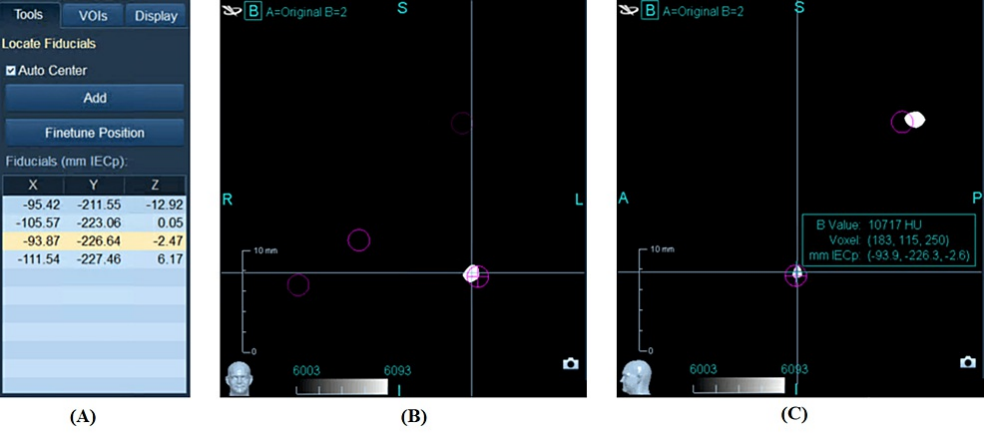
Comparison of fiducial locations in each breathhold image. (A) Three-dimensional coordinate locations of the four fiducials in the reference normal exhale breathhold scan, (B) coronal view of the third fiducial in the exhale scan, (C) sagittal view of the same fiducial. In (B) and (C), the circles depict the specified fiducial location in the reference normal exhale scan, and the white marks are the visible fiducial locations in the fused normal inhale scan.

Fiducial agreement in the breathhold scans of the pancreatic cancer patient can be seen in Figures 4A-4C and Table 1. In the first three scans, one exhale matched the inhale, but the superior-most fiducial of the other exhale scan mismatched, therefore one additional normal exhale scan was acquired. The comparison of the final three scans in Table 1 suggests that there was likely to be less than a millimeter of deformation of the tumor in the vicinity of the fiducials.

**Table 1:** Comparisons of fiducial locations to the reference normal exhale and normal inhale scans. The table shows that the patient had moved during the second exhale scan, so it was re-scanned, and then both the inhale and exhale matched the reference exhale to within 1mm. Fiducials are numbered from cranial to caudal within the patient and the coordinates are with respect to the DICOM origin on the planning CT after fusing images. DICOM: Digital Imaging and Communications in Medicine

FID	Best exhale, reference (mm)			Rejected exhale (mm)				Inhale (mm)				Re-scanned exhale (mm)			
	X	Y	Z	X	Y	Z	3D Err	X	Y	Z	3D Err	X	Y	Z	3D Err
1	-95.4	-211.6	-12.9	-95.9	-213.7	-10.7	3.1	-94.8	-211.3	-13.4	0.8	-95.6	-211.8	-12.7	0.4
2	-105.6	-223.1	0.1	-105.0	-222.9	-0.5	0.8	-105.4	-222.9	-0.4	0.5	-105.4	-223.4	0.0	0.4
3	-93.9	-226.6	-2.5	-93.5	-226.4	-2.7	0.5	-94.5	-226.3	-2.6	0.7	-94.0	-226.3	-2.7	0.4
4	-111.5	-227.5	6.2	-111.2	-226.6	6.1	0.9	-111.1	-227.2	6.4	0.6	-111.5	-227.6	6.2	0.1

## Discussion

Although studies regarding Synchrony [7,8] and 4DCT [9,10] began to be published for SBRT at about the same time, there are now about 10 times more PubMed-indexed papers for 4DCT as of June 2022, with 665 hits for "radiation AND lung AND 4DCT" while only 49 for "radiation AND lung AND Synchrony." Similarly, there are more than 400 instances of "radiation AND 'deep inspiration breath hold'." Since Synchrony with breathhold scans has achieved remarkably good results, to be fair, it is important to ensure that an equitable number of publications of this technique is also permitted [5]. Accuray recommends normal exhale and normal inhale breathhold scans for Synchrony motion tracking, but since not all users employ that technique, it is important for researchers to specify which CT scan technique was used when reporting their results; these treatment variables are needed to enable QUANTEC and HyTEC style initiatives [11,12].

Deep inspiration breathhold (DIBH) is different than normal breathhold [3]. When patients are first instructed to take a normal breathhold, they often instinctively take a deep breathhold instead, and some explanation and practice are often needed until they can hold their breath in a normal position. Patients will be breathing normally during Synchrony treatment, which tracks the tumor motion while the radiation beam is on. Therefore, DIBH should not be used during the CT scan, since patients will not need to breathe deeply during treatment. For the normal exhale position, the simplest instructions that we have found to work well are as follows: "take a deep breath in..." "blow it out, ..." "...and hold it." Similar instructions are practiced for the normal inhale, like: "take a deep breath in..." "let out a little bit, ..." "...and hold it." Instead of giving the patient a theoretical description of DIBH versus "normal breathhold," it may be easier to give simple adjustment guidance like "just relax a little more," or "blow out a tiny bit more," etc. since we can see the breathing waveform on the RPM system. The normal breathhold must be practiced interactively with the patient until they can do it reliably, before performing the planning CT scans [3].

Early in our experience with these techniques, we had to rescan an exhale phase for one patient and an inhale phase for another. After we gained the skill, it has become quite infrequent to need to rescan any of the breathhold phases, and we are approaching a level where we can often tell by watching the patient and the slices during the scan whether it worked well or not. Nevertheless, we continue to perform a quick fusion while the patient remains on the CT table, just in case more scans are needed. As the patient is getting up from the table, we continue to refine the fusion and usually have it finalized by the time the patient leaves the department; thus far no patient has needed to be called back for a rescan. The case study in Figures 4A-4C and Table 1 was selected because it was one of the only recent cases that needed to have one scan repeated.

Although only a single CT scan is needed for Synchrony, none of the validation in the previous paragraph would have been possible if we only had a single scan [2,3]. The main advantage of using multiple scans is that they can be compared to each other, fiducial to fiducial, to ensure the observed deformation and residual motion in the vicinity of the fiducials is acceptable. Particularly, the exhale scan is important since patients are in the exhale position most of the time. Therefore, we use two normal exhale breathhold scans and an additional normal inhale breathhold scan. Theoretically, the two exhale scans should match each other “exactly,” but if we find a large discrepancy, we will rescan one as in Table 1. After choosing one exhale scan as the planning CT, any residual deformation or patient motion observed in the other two scans can be accounted for in the contours, or as an additional margin [2,3].

A previous study recommended a 3mm additional margin to account for apparent deformation of the tumor as seen by fiducials [2]. (Coincidentally, the discrepancy in the superior-most fiducial in the second exhale scan of Table 1 was also about 3mm.) The 3mm additional margin would have been sufficient to accommodate this uncertainty, but by fusing and comparing the multiple breathhold scans, it might become possible to overcome the need for additional margin specifically to accommodate deformation, although some amount of margin is still advisable for many other reasons. Future studies should investigate this in more detail. For now, we are merely pointing out that the normal breathhold scans worked for our institution.

Thin slices are recommended by Accuray to improve the quality of the digitally reconstructed radiograph (DRR) and thus improve targeting accuracy. Our scanner has options for 0.625mm, which we use for cranial targets, and 1.25mm slices, which we use for most extracranial targets. In our initial testing of our CT scanner, we were able to achieve faster scans that handled patient motion better with 1.25mm slices than 0.625mm. Furthermore, to enable inferior/superior entrance angles it is preferable to scan at least 15cm above and below the target, and only 512 slices are allowed in the Precision planning system, implying that 0.625mm slices would not have provided much additional access. With more exhaustive testing of 0.625mm versus 1.25mm slice thickness, it might be possible to alleviate some of the 4DCT artifacts. However, the problems in Figures 2A, 2B and Figures 3A-3C are much larger than a millimeter, so the use of submillimeter slice thickness does not seem likely to solve it all, but it could be a potential future area of investigation.

**Figure 5:**
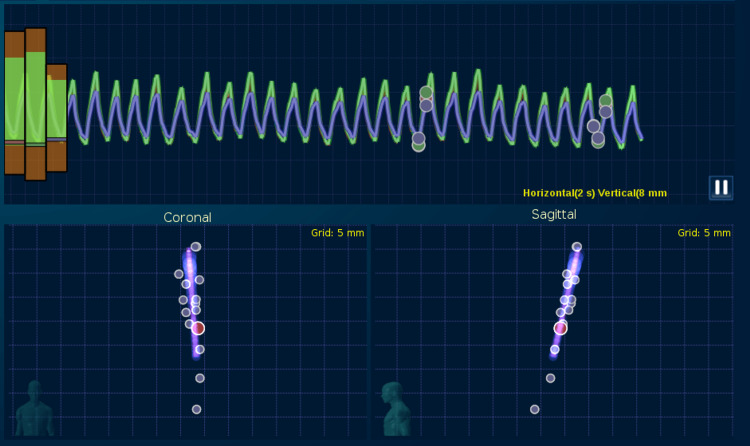
Some breathing irregularities in at-rest adults can be accommodated by the Synchrony tracking system, but it might explain some of the challenges encountered when using 4DCT. From the coronal and sagittal views, it can be seen that it is not uncommon for exhale and inhale excursions 5mm or more beyond the bulk of the breathing patterns to occur.

Soon after the inception of 4DCT [13-15], it was investigated for SBRT [9,10]. One of the earliest studies used a phantom consisting of a pear shape, several spheres of various sizes, and a triangle oriented horizontally [15]. The coronal plane showed abrupt discontinuities in the diagonal edge of the triangle, indicating that some slices were not in the ideal sequence within an appropriate breathing phase, or that target motion occurred during the gantry rotation within the slices (Figs 5-8 in the study by Pan et al. [15]). Furthermore, in some subfigures of that study, the pear shape looked more like a sphere and some of the spheres looked more like a pear shape, and some of the objects appeared to separate into pieces in some phases and re-join in other phases. If any type of CT with artifact was used for treatment planning, it could degrade the results regardless of tracking method. Therefore, artifact reduction algorithms were promptly published [16,17] but subsequent articles continued to note similar characteristic artifacts in excess of several millimeters [18-21]. In general, the 4DCT artifacts were noted to have proportionately more effect for faster breathing [18], for regions of larger target motion [18], and for smaller target size [21]. Conceptually, a 4DCT with a lot more than 10 phases might look much better, but may still have practical limitations. Online adaptive planning for MRI-guided systems and Ethos are also possible methods [22,23]. Advanced algorithms continue to be published up to the present day for potentially improved methods, but some work would be needed to prove whether they could achieve the submillimeter specification or not [19,24-27].

For linac cases, the 4DCT is usually done prior to treatment, whereas the Synchrony algorithm adapts to the patient’s breathing during the treatment. Normal adult restful breathing is from 12-20 bpm [6] and a 4DCT typically takes 3-5 min to acquire, so the expected number of breaths per 4DCT scan is about 30-100. The 4DCT is usually performed several days or weeks prior to treatment. In contrast, the Synchrony model is updated adaptively during each day of treatment. Synchrony first acquires stereoscopic kilovoltage x-ray images at the peak and valley of patient breathing, then takes about 10 more stereoscopic images in rapid succession, and then takes three more images timed to fill in any potential gaps in the breathing cycle, for a total of 15 images in the model (Figure 5). During treatment, Synchrony takes a burst of three images every 60 s by default, so a 15-min treatment delivery would correspond to 45 additional images to enable the model to adapt to the patient's breathing changes. If at any time during treatment the algorithm or user detects any anomalies, the treatment can be paused and images taken manually to rebuild the model corresponding to the patient's latest breathing pattern.

Variations in patient breathing, from one cycle to the next, are one of the challenges that 4DCT would need to overcome since multiple breaths are combined into one 4DCT scan. Synchrony models are not perfect either, although several parameters such as rigid body errors are computed throughout the treatment to enable users to set acceptable performance tolerances [28]. The dots in the upper section of Figure 5 illustrate that the Synchrony algorithm attempts to acquire images at the peak and valley of breathing and at some locations in between. The measured physical position of the three “light emitting diodes” on the anterior chest of the patient is plotted as breathing waveforms in the upper section of Figure 5 showing that the peak and valley extents of each breath can be different than the previous one. It can be seen that Synchrony can handle this well because all the tumor position dots in the lower portion graphs show consistency with the overall model, even in situations like Figure 5 where the coronal and sagittal views reveal 5mm or more variation in the tumor position at the extents of exhale and inhale. Not all 4DCT scanners handle non-systematic variations in target position well, which is why 4DCT may need advanced algorithms [19,24-27]. In the meantime, normal breathhold scans combined with Synchrony tracking can accommodate these variations during treatment.

Some patients cannot hold their breath for long, but it is not necessary for them to hold their breath for the entire scan. With precisely timed coaching accounting for the processing time of the CT scanner, it is possible to watch the slices appearing on the console and give patients breathhold instructions as the scan is already beginning. In this way, they only need to hold their breath while the tumor itself is being scanned, plus a few centimeters superior and inferior for safety margin. Therefore, the fiducials most likely to mismatch are the superior-most and inferior-most ones, as in Table 1, since the patient is less likely to be fully stable at those portions of the scan.

The main limitation of this study is that it is based on several cases and a phantom test; therefore proving the best technique is beyond the scope of this study. We merely present a few examples and show the feasibility of Accuray’s recommendation of normal exhale and normal inhale breathhold scans, combined with Synchrony live tracking.

## Conclusions

When combined with Synchrony live motion tracking, we have found normal exhale and normal inhale breathhold scans to be feasible and effective, as compared to 4DCT scans. We know that the fiducial, phantom ball, and tumor do not separate and re-join as they appear to in some 4DCT scans; it is most likely that this apparent deformation is due to 4DCT artifact. By examining two normal breathhold exhale scans to an inhale scan, we verified that the amount of tumor deformation and residual motion in the vicinity of the fiducials in those scans was acceptably small.
